# Violent Operation of the Eau Medicinale D'Husson

**Published:** 1810-11

**Authors:** 


### *Violent Operation of the Eau Medicinale D'Husson.*

### *For the Medical Journal.*

THE following instance of the violent action of a nostrum called *Eau Medicinale D'Husson*, is communicated to the Editors, by ROBERT ADAMS, Esq. Surgeon, Charlotte-street, Fitzroy Square.

---

ABOUT the 18th of December last Mr. Adams was called to a person who had taken the *Eau Medicinale*, and who was then suffering under most alarming symptoms of its effects. The patient, a Fishmonger near St. Martin's-lane, had taken two doses of the nostrum: these produced syncope, cold sweats, extreme prostration of strength, excessive evacuations from the stomach and bowels, accompanied with a pulse scarcely perceptible, and a degree of insensibility that indicated the approach of death. By the active employment of the most powerful cordials, his dissolution was, however, prevented: but he is still suffering from the operation of the remedy.

It is proper to remark that this person was, at the time of taking the *Eau Medicinale*, an athletic man, subject occasionally to be disordered with regular gouty paroxysms, the disease always appearing in the extremities. When he took the medicine he had gout in his feet only, but on the 20th, two days after its administration, the disease appeared, with unusual severity, in his hands and head.

There are circumstances of great importance in this case. The remedy endangered the patient's life, and his disease was seriously encreased; and now, ten months after taking the nostrum, his system remains in a state of great debility.

*October* 17, 1810.

